# Trends in Palliative Care, Hospice Utilization, and Outcomes in Hospitalized Pancreatic Cancer Patients: A Nationwide Analysis

**DOI:** 10.7759/cureus.29351

**Published:** 2022-09-20

**Authors:** Kuldeepsinh P Atodaria, Steven J Cohen, Samyak Dhruv, Shravya R Ginnaram, Shreeja Shah

**Affiliations:** 1 Internal Medicine, Abington Jefferson Health, Abington, USA; 2 Medical Oncology, Thomas Jefferson University Hospital, Philadelphia, USA; 3 Internal Medicine, MedStar Health, Washington DC, USA

**Keywords:** outcomes, national inpatient sample, cancer complications, hospice, palliative care, mortality, pancreatic cancer

## Abstract

Background and objective

The prognosis of pancreatic cancer (PC) is generally poor. PC responds only modestly to chemotherapy and chemoradiation, and surgical resection remains the only curative option. The risk of recurrence is high. PC patients are encountered in the hospital on initial diagnosis and later for surgeries and complications from PC. We analyzed PC hospitalizations in the United States as reported in the National Inpatient Sample (NIS) database from 2005 to 2011 to determine the extent to which aggressive interventions could be avoided, thereby decreasing the cost of hospitalization. We analyzed trends in palliative care utilization and hospice services.

Methods

The International Classification of Disease 9th Revision (ICD-9) codes were used to identify diagnoses and procedures performed. Weighted analysis was performed using SPSS Statistics 28.0 (IBM Corp., Armonk, NY). Dispositions at discharge were noted. Complications and procedures performed were also documented.

Results

A total of 574,522 cases with PC were identified. Trends are reported chronologically (2005 to 2011). Over time, inpatient deaths for PC have decreased (11.2%, 11.1%, 9.8%, 9.8%, 9.5%, 8.4%, 8.1%; p<0.001), and hospice discharges (HD) have increased (10.2%, 11.4%, 11.4%, 12,2%, 12.6%, 12.4%, 12.7%; p<0.001). Palliative care utilization has increased (2.9%, 3.9%, 3.8%, 5.6%, 8.8%, 10.2%, 11.9%; p<0.001). Complications including peritonitis, thrombosis, hypovolemia/shock, and acute kidney injury (AKI) have increased mortality rates and HD.

Conclusion

There is an increasing trend of palliative care and hospice service utilization among hospitalized PC patients. Until better-targeted treatments and screening become available, mortality and morbidity will remain high. The proportion of patients receiving aggressive interventions remains high and is associated with poor outcomes. It is desirable to conduct palliative care evaluation (PCE) early in patients with advanced disease and avoid aggressive interventions.

## Introduction

Pancreatic cancer (PC) is a malignancy with high mortality and a five-year survival rate of around 4-6% [1,2]. The incidence of PC has been rising with each passing year [2]. PC is often diagnosed in the late stages of the disease, as it is often asymptomatic in the early stages [3]. The only curative option is surgical resection of resectable tumors [2,4]. PC is not very responsive to chemotherapy and radiation therapy. Thus, advanced unresectable tumors inevitably have a poor prognosis. This is reflected by the fact that the five-year mortality of PC closely parallels its incidence [1,5]. Even among patients who are cured by surgery, the rate of recurrence is high, with five-year survival ranging from 15 to 25% [5-7]. PC patients are encountered in the hospital on initial presentation with nonspecific symptomatic disease and later for surgeries or due to complications arising from the treatments or disease progression. Frequent hospitalizations and re-hospitalizations indicate poor quality of care for patients with advanced cancers [8,9]. Indicators of aggressive care near death include chemotherapy, ED visits, hospitalization (including death during hospitalization), and ICU admissions [10-15]. PC is one of the leading cancers in terms of frequency of ED visits and hospitalizations in the last six months and last two weeks of life [9]. Palliative care consultation is associated with less aggressive care near the end-of-life (EOL) in patients with PC [10,16]. Palliative care interventions are associated with improved quality of life and satisfaction near EOL [16-21].

## Materials and methods

Study design

In this cross-sectional retrospective analysis, we analyzed PC hospitalizations reported in the National Inpatient Sample (NIS) database from 2005 to 2011 to find out what aggressive interventions could be avoided in patients, thereby decreasing hospitalization costs and increasing the utilization of palliative care and hospice services.

Primary and secondary outcomes

The study's primary outcome was the disposition of the patient at discharge from the hospital. We classified patients into three groups based on their discharge disposition: died, hospice discharge (HD), and other discharges (which included all other discharges). The secondary outcomes of the study were the complications arising from PC and interventions performed during the hospitalization, as mentioned below under Methods. 

Methods

NIS is an administrative database, a part of the Healthcare Cost and Utilization Project (HCUP). NIS is the largest publicly available all-payer inpatient healthcare database designed to generate US regional and national estimates of inpatient utilization, access, cost, quality, and outcomes. Unweighted, it contains data from more than seven million hospital stays each year. Weighted, it estimates more than 35 million hospitalizations nationally. NIS consists of de-identified patient information that is nationally available, which was submitted for the Institutional Review Board (IRB) review. The Thomas Jefferson University Hospital IRB determined that our study did not require IRB approval and permitted us to proceed with the analysis.

Adult cases with the International Classification of Disease 9th Revision (ICD-9) diagnosis codes for PC were identified from the database for the years 2005 to 2011. Furthermore, ICD-9 diagnosis codes were used to identify patients who had metastases of various organs and lymph nodes. ICD-9 procedural codes were used to identify cases with diagnostic procedures such as laparoscopy, biopsies, and other diagnostic procedures of the abdomen, including endoscopic retrograde pancreatography. Treatments and interventions were identified using ICD-9 procedure codes for various forms of pancreatectomy, and chemotherapy administration. Common aggressive interventions including transfusion of red cells and other blood products, intravenous anticoagulation, thrombolytics, vasopressors, an inferior vena cava filter (IVCF) insertion, mechanical ventilation (MV), dialysis, and parenteral nutrition were identified. Common complications arising from PC such as deep vein thrombosis (DVT), pulmonary embolism (PE), peritonitis, and complications relating to the stomach, duodenum, gall bladder, biliary system, and pancreas were identified. Other complications such as cachexia, hypovolemia, shock, acute kidney injury (AKI), and depression were also identified. In addition, cases that received palliative care evaluation were identified. The codes used to identify these conditions can be reviewed in Appendix 1.

Charlson Comorbidity Index (CCI) was calculated using a combination of indicators for chronic conditions as reported in the NIS database and ICD-9 diagnosis codes. CCI is a well-validated measure of comorbidity used to predict one-year mortality in patients [22,23].

Statistical analysis

SPSS Statistics for Windows, Version 28.0 (IBM Corp., Armonk, NY) was used for data analysis. Chi-square tests were utilized to check the association between different categorical variables of interest and outcomes of the hospitalizations. Mann-Whitney U tests were used to test the association between non-normally distributed continuous variables, and median values were reported. Trends were checked using the Cochran-Armitage test of trend. For the values reported in the tables, all p-values were significant (p<0.05) unless otherwise stated. The non-significant values are marked with an asterisk (*). All the results are statistically weighted using DISCWT (weight of discharges in the universe) as provided by HCUP in the NIS database.

## Results

A total of 574,522 cases of PC were identified. Of those, 55,095 (9.6%) patients died during the hospital stay, with 68,497 (11.9%) HD; 293,747 (51.1%) of the cases had metastatic disease. Metastatic disease was associated with increased mortality compared to cases without metastasis (11.8% and 7.3%, respectively, p<0.001).

Mortality and HD increased consistently with age, as seen in Table 1. As for sex distribution, 50.5% were male and 49.5% female. Males had higher mortality than females (10.2% vs. 9%, p<0.001), while females had higher HD (12.5% in females and 11.3% in males, p<0.001). The mean age at admission for cases that died was 69.50 years (SD: 12.20), and the mean age for those with HD was 71.18 years (SD: 12.43). The mean CCI was 5.34 (SD: 3.04) for cases that died and 5.59 (SD: 2.97) for HD.

**Table 1:** Discharge outcomes in pancreatic cancer patients based on various demographic characteristics P-values for all numbers reported were <0.001 unless otherwise stated and indicated by *. The numbers/percentages may not add up to the total number/100% as some of the values may have been missing/unreported in the original database SD: standard deviation

Characteristic	Frequency (n)	Percentage (%)	Disposition at discharge					
			Died		Hospice		Other discharge destinations	
			Frequency	Percentage	Frequency	Percentage	Frequency	Percentage
Total	574,522	100%	55,095	9.60%	68,496	11.90%	450,931	78.50%
Mean age (SD)			69.50 (12.20)	P<0.001	71.18 (12.43)	P<0.001	67.34 (12.52)	P<0.001
Age group, years								
18-30	1,827	0.30%	42	2.30%	57	3.10%	1,728	94.60%
31-40	7,691	1.30%	455	5.90%	483	6.30%	6,753	87.80%
41-50	40,614	7.10%	3,036	7.50%	3,407	8.40%	34,171	84.10%
51-60	108,740	18.90%	9,791	9%	10,090	9.30%	88,859	81.70%
61-70	159,772	27.80%	14,955	9.40%	16,863	10.60%	127,955	80.10%
71-80	156,098	27.20%	15,558	10%	19,607	12.60%	120,932	77.50%
81-90	88,472	15.40%	9,791	11.10%	15,557	17.60%	63,124	71.30%
>91	11,309	2%	1,467	13%	2,432	21.50%	7,409	65.50%
Sex								
Male	289,793	50.50%	29,491	10.20%	32,891	11.30%	227,410	78.50%
Female	284,548	49.50%	25,577	9%	35,605	12.50%	223,366	78.50%
Race								
Caucasian	355,115	74.70%	32,537	9.20%	42,170	11.90%	280,408	79%
Black	58,183	12.20%	6,633	11.40%	7,260	12.50%	44,290	76.10%
Hispanic	34,826	7.30%	3,330	9.60%	3,417	9.80%	28,079	80.60%
Asian/Pacific Islander	13,389	2.80%	1,596	11.90%	636	4.80%	11,157	83.30%
Native American	1,897	0.40%	195	10.30%	208	11%	1,494	78.80%
Other	12,148	2.60%	1,291	10.60%	1,421	11.70%	9,436	77.70%
Type of insurance								
Medicare	328,669	57.30%	30,164	9.20%	43,346	13.20%	255,158	77.60%
Medicaid	39,536	6.90%	3,476	8.80%	4,147	10.50%	31,912	80.70%
Private	176,932	30.90%	17,166	9.70%	16,625	9.40%	143,142	80.90%
Self-pay	12,772	2.20%	1,403	11%	1,410	11%	9,959	78%
No charge	1,607	0.30%	158	9.80%	242	15.10%	1,207	75.10%
Other	13,915	2.40%	2,516	18.10%	2,563	18.40%	8,836	63.50%
Charlson Comorbidity Index (CCI)								
Mean CCI (SD)			5.34 (3.04)*	P=0.051	5.59 (2.97)	P<0.001	5.34 (2.90)	P<0.001
Hospital location/teaching status								
Rural	45,431	9.10%	5,974	13.10%	5,560	12.20%	33,896	74.60%
Urban nonteaching	185,826	37.20%	18,745	10.10%	25,766	13.90%	141,315	76%
Urban teaching	268,399	53.70%	22,196	8.30%	28,505	10.60%	217,698	81.10%
Admission type								
Emergent	278,492	54.70%	28,756	10.30%	43,894	15.80%	205,843	73.90%
Urgent	110,633	21.70%	10,480	9.50%	15,349	13.90%	84,805	76.70%
Elective	119,054	23.40%	8,807	9.50%	9,002	7.60%	101,245	85%
Trauma center	670	0.10%	78	11.60%	100	14.90%	492	73.40%

We analyzed the impact of the primary site of PC on in-hospital outcomes. The primary part of the pancreas was not specified for 49.3% of the cases. For the cases in which it was specified, the pancreatic head was the most commonly reported location of primary PC (159,557 cases, 27.8%). The effect of the site of primary PC on death in the hospital and HD can be reviewed in Table 2. 

**Table 2:** Distribution of pancreatic cancer cases based on the location of the primary tumor and their discharge outcomes P-values for all the numbers reported were <0.001 unless otherwise stated

Location of the primary tumor	Frequency (n)	Percentage (%)	Died		Hospice		Other discharge destinations	
			Frequency	Percentage	Frequency	Percentage	Frequency	Percentage
Head of pancreas	159,557	27.80%	9,648	6%	14,725	9.20%	135,184	84.70%
Tail of pancreas	33,127	5.80%	2,526	7.60%	4,072	12.30%	26,529	80.10%
Body of pancreas	20,940	3.60%	1,297	6.20%	2,202	10.50%	17,441	83.30%
Duct of pancreas	6,862	1.20%	451	6.60%	632	9.20%	5,779	84.20%
Islets of Langerhans	5,329	0.90%	258	4.80%	185	3.50%	4,886	91.70%
Other	65,441	11.40%	7,161	10.90%	8,120	12.40%	50,161	76.70%
Unspecified part of pancreas	283,267	49.30%	33,754	11.90%	38,561	13.60%	210,952	74.50%
Total	574,552	100%	55,095	9.60%	68,497	11.90%	450,932	78.50%

We next looked at the site of metastases and their relationship to in-hospital outcomes, as reported in Table 3. Notable findings are as follows. Metastases were associated with increased HD compared to non-metastatic PC cases (15.6% with metastases and 8.1% without metastases, p<0.001). The liver was the most common site of metastasis (33.5%). Liver metastases were associated with increased death in the hospital (12.0% with liver metastases and 8.4% without metastases, p<0.001) and increased HD (16.2% with liver metastases and 9.6% without liver metastases, p<0.001). Metastases of other abdominal organs were reported in 13.8% of the cases. Other sites of metastasis with a sizeable frequency were the thoracic cavity [including lungs (7.9% for lungs only), mediastinum, pleura, and other respiratory organs; 8.7% total], and bone/bone marrow (3.4%). Thoracic cavity metastasis was associated with increased death in the hospital (14.6% with thoracic metastases and 9.1% without thoracic metastases, p<0.001) and increased HD (17.9% with thoracic metastases and 11.4% without thoracic metastases, p<0.001). Bone metastasis was also associated with increased death in the hospital (14.6% with bone metastases and 9.4% without bone metastases, p<0.001) and increased HD (18.2% with bone metastases and 11.7% without bone metastases, p<0.001). Brain and spinal cord (CNS) metastases were reported in only 0.6% of the cases but were associated with higher deaths in the hospital (17.2% with CNS metastases and 9.5% without CNS metastasis, p<0.001) and increased HD (21.7% with CNS metastases and 11.9% without CNS metastases, p<0.001).

**Table 3:** Distribution of patients based on site of metastasis and its effect on discharge outcomes *Marked values had non-significant p-values (p>0.05), and the rest of the values were significant (p<0.05). **Lymph node numbers are not included in the "Total metastases". Lymph node numbers may have been under-reported in the database, leading to a low percentage (7.8%) having diagnosis codes for it

Location of metastatic disease	Frequency (n)	Percentage (%)	Died		Hospice		Other discharge destinations	
			Frequency	Percentage	Frequency	Percentage	Frequency	Percentage
Liver	192,218	33.50%	23,005	12%	31,676	16.50%	137,537	71.60%
Abdominal metastasis (duodenum, small intestine, colon, rectum, spleen, kidneys, adrenals, spleen, other abdominal organs; liver not included)	79,144	13.80%	8,966	11.30%	12,500	15.80%	57,678	72.90%
Lung	45,588	7.90%	6,549	14.40%	8,207	18%	30,832	67.60%
Thoracic cavity (lung, mediastinum, pleura, other respiratory organs)	50,121	8.70%	7,312	14.60%	8,960	17.90%	33,849	67.50%
Brain and spinal cord	3,728	0.60%	636	17.10%	809	21.70%	2,282	61.20%
Bone/bone marrow	19,289	3.40%	2,819	14.60%	3,507	18.20%	12,962	67.20%
Other nervous system-related	367	0.10%	43*	11.7%*	92	25%	232	63.20%
Adrenal glands	4,093	0.70%	500	12.20%	750	18.30%	2,843	69.40%
Metastasis to breast	134	0%	29	21.60%	23*	17.2%*	82	61.20%
Ovary	1,184	0.20%	64	5.40%	168	14.20%	951	80.40%
Kidney	1,896	0.30%	241	12.70%	344	18.10%	1,311	69.10%
Other organs of the urinary system	946	0.20%	118	12.50%	136	14.40%	692	73.20%
Genitals	744	0.10%	64*	8.6%*	119	16%	560	75.30%
Other unspecified sites of metastasis	19,870	3.50%	1,815	9.10%	2,340*	11.8%*	15,715	79.10%
Total metastases	293,747	51.10%	34,678	11.80%	45,805	15.60%	213,264	72.60%
Cases without metastasis	280,775	48.90%	20,417	7.30%	22,691	8.10%	237,667	84.60%
Positive lymph nodes**	44,627	7.80%	2,955	6.60%	3,837	8.60%	37,835	84.80%

We also looked into the impact of other medical issues and complications on in-hospital outcomes, which can be reviewed in Table 4. Notable results are as follows. DVT and PE were reported in 5.4% and 4% of cases, respectively. DVT was associated with increased death in the hospital (11.5%) compared to cases without DVT (9.5%, p<0.001), and increased HD (16.4% with DVT and 11.7% without DVT, p<0.001). PE was associated with increased death in the hospital (13.6%) compared to cases without PE (9.4%, p<0.001), and increased HD (16.1% with PE and 11.7% without PE, p<0.001). IVCF insertion was reported in 2% of the total PC cases, with no significant difference in deaths in the hospital (9.7% with IVCF insertion and 9.6% without IVCF insertion, p=0.676). IVCF insertion was associated with higher HD (16.8% with IVCF insertion and 11.8% without IVCF insertion, p<0.001). Oral anticoagulation was reported in 4.2% of cases and was associated with decreased death in the hospital (6.7% with anticoagulation and 9.7% without anticoagulation, p<0.001) and increased HD (12.6% with anticoagulation and 11.9% without anticoagulation, p<0.001). Mechanical ventilation (MV) was reported in 3.1% of cases, with higher deaths in the hospital (51.7% with MV and 8.2% without MV, p<0.001) but low HD (7.8% with MV and 12.1% without MV, p<0.001). The associations with other interventions can be reviewed in Table 5.

**Table 4:** Effect of complications of pancreatic cancer on discharge outcomes *Indicates p-value of >0.05 (non-significant); all other reported numbers had p<0.001 The conditions included under the grouped complications (Cx) can be found in Appendix 1 DVT: deep vein thrombosis; PE: pulmonary embolism; AKI: acute kidney injury

Characteristic	Frequency (n)	Percentage (%)	Disposition at discharge					
			Died		Hospice		Other discharge destinations	
			Frequency	Percentage	Frequency	Percentage	Frequency	Percentage
Total	574,522	100%	55,095	9.60%	68,496	11.90%	450,931	78.50%
Metastasis	293,747	51.10%	34,678	11.80%	45,805	15.60%	213,264	72.60%
Gastroduodenal complications (Cx)	27,690	4.80%	2,404	8.70%	3,910	14.10%	213,376	77.20%
Gall bladder-related (Cx)	15,129	2.60%	655	4.30%	805	5.30%	13,668	90.30%
Biliary system-related (Cx)	112,533	19.60%	6,952	6.20%	12,550	11.20%	93,031	82.70%
Pancreas-related (Cx)	57,838	10.10%	3,385	5.90%	5,551	9.60%	48,902	84.50%
Peritonitis	10,902	1.90%	1,790	16.40%	1,350*	12.4%*	7,763	71.20%
DVT	31,238	5.40%	3,582	11.50%	5,128	16.40%	22,528	72.10%
PE	22,939	4%	3,124	13.60%	3,699	16.10%	16,116	70.30%
Depression	37,430	6.50%	2,273	6.10%	4,538*	12.1%*	330,619	81.80%
Cachexia	18,763	3.30%	3,159	16.80%	4,112	21.90%	11,491	61.20%
Hypovolemia/shock	25,009	4.40%	8,620	34.50%	3,198	12.80%	13,191	52.70%
AKI	58,946	10.30%	16,058	27.20%	10,318	17.50%	32,571	55.30%

**Table 5:** Effect of procedures/interventions on discharge outcomes for pancreatic cancer patients *Indicates p-value of >0.05 (non-significant); all other reported numbers had p<0.001. **For procedures that are grouped, e.g., diagnostic procedures, please refer to Appendix 1 for more details

Procedure/intervention	Frequency (n)	Percentage (%)	Disposition at discharge					
			Died		Hospice		Other discharge destinations	
			Frequency	Percentage	Frequency	Percentage	Frequency	Percentage
Total	574,522	100%	55,095	9.60%	68,496	11.90%	450,931	78.50%
Diagnostic procedures	51,674	9%	2,201	4.30%	4,045	7.80%	45,428	87.90%
Chemotherapy	18,996	3.30%	2,058	5.60%	1,154	6.10%	16,784	88.40%
Neutropenia	7,522	1.30%	547	7.30%	539	7.20%	6,436	85.60%
Pancreatic surgeries								
All pancreatic resection surgeries	48,406	8.40%	1,969	4.10%	224	0.50%	46,214	95.50%
Whipple procedure	33,614	5.90%	1,555	4.60%	157	0.50%	31,902	94.90%
Total pancreatectomy	2,069	0.40%	115	5.60%	5	0.20%	1,949	94.20%
Subtotal pancreatectomy	460	0.10%	5	1.10%	13	2.80%	442	96.10%
Proximal pancreatectomy	468	0.10%	29	6.20%	0	0%	439	93.80%
Distal pancreatectomy	10,459	1.80%	213	2%	44	0.40%	10,202	97.50%
Other pancreatectomies	1,661	0.30%	104	6.30%	5	0%	1,552	93.40%
Bile stent	41,927	7.30%	1,467	3.50%	3,955	9.40%	36,505	87.10%
Sphincter of Oddi-related procedures**	50,168	8.70%	1,840	3.70%	4,854	9.70%	43,474	86.70%
Transfusion of blood products**	98,961	17.20%	11,176	11.30%	12,185	12.30%	75,600	76.40%
Transfusion of red cells	96,262	16.80%	10,610	11%	11,730	12.20%	73,923	76.80%
Injected anticoagulants	6,075	1.10%	458	7.50%	508	8.40%	5,109	84.10%
Injection of thrombolytic	1,577	0.30%	205	13%	181*	11.5%*	1,191	75.50%
Long-term anticoagulation	24,121	4.20%	1,616	6.70%	3,045	12.60%	19,460	80.70%
IVC filter	11,460	2%	1,112*	9.7%*	1,929	16.80%	8,419	73.50%
Mechanical ventilation (MV)								
Total MV	17,776	3.10%	9,187	51.70%	1,393	7.80%	7,196	40.50%
<96 hours	11,974	2.10%	6,248	52.20%	925	7.70%	4,801	40.10%
>96 hours	5,784	1%	2,931	50.70%	468	8.10%	2,385	41.20%
Unspecified duration	18	0%	8	44.40%	0	0%	10	55.60%
Dialysis	1,213	0.20%	175	14.40%	175	14.40%	863	71.20%
Injection of vasopressors	1,890	0.30%	854	45.20%	225*	11.9%*	811	42.90%
Parenteral nutrition	28,454	5%	3,407	12%	3,212	11.30%	21,834	76.70%
Palliative care evaluation	40,327	7%	14,520	36%	12,811	31.80%	12,996	32.20%

AKI (reported in 10.3% of cases) was associated with increased death in the hospital (27.2% with AKI and 7.6% without AKI, p<0.001) and increased HD (17.5% with AKI and 11.3% without AKI, p<0.001). Hypovolemia/shock (4.4% cases) was associated with increased death in the hospital (34.5% with hypovolemia/shock and 8.5% without hypovolemia/shock, p<0.001) and increased HD (12.8% with hypovolemia/shock and 11.9% without hypovolemia/shock, p<0.001). Cachexia (3.3% cases) was associated with increased death in the hospital (16.8% with cachexia and 9.3% without cachexia, p<0.001) and increased HD (21.9% with cachexia and 11.6% without cachexia, p<0.001). Other complications can be reviewed in Table 4.

A total of 48,406 cases (8.4%) were hospitalized for surgical intervention (Table 5). Surgical intervention was associated with decreased death in the hospital (4.1% with surgery and 10.1% without surgery, p<0.001) and decreased HD (0.5% with surgery and 13.0% without surgery, p<0.001); 7.3% of the total cases received an endoscopic biliary stent placement. Biliary stent placement was associated with decreased death in the hospital (3.5% with the stent and 10.1% without the stent, p<0.001) and decreased HD (9.4% with the stent and 12.1% without the stent, p<0.001). Chemotherapy was reported in 3.3% of cases and was associated with decreased death in the hospital (5.6% with chemotherapy and 9.7% without chemotherapy, p<0.001) and decreased HD (6.1% with chemotherapy and 12.1% without chemotherapy, p<0.001).

Palliative care evaluation (PCE) was reported in 7% of the cases. PCE was associated with increased death in the hospital (36% with PCE and 7.6% without PCE, p<0.001), and increased HD (31.8% with PCE and 10.4% without PCE, p<0.001). The remainder of the numbers for other therapeutic interventions can be seen in Table 5. 

The number of PC hospitalizations has been increasing with each passing year (Table 6). Inpatient deaths for PC decreased from 2005 to 2011 (11.2%, 11.1%, 9.8%, 9.8%, 9.5%, 8.4%, 8.1%, p<0.001), while HD increased in the same period (10.2%, 11.4%, 11.4%, 12,2%, 12.6%, 12.4%, 12.7%, p<0.001). The utilization of palliative care increased from 2005 to 2011 (2.9%, 3.9%, 3.8%, 5.6%, 8.8%, 10.2%, 11.9%, p<0.001). The proportion of hospitalized PC patients with metastatic disease decreased from 2005 to 2011 (52.6%, 50.5%, 52.8%, 50.9%, 51%, 50.6%, 50%, p<0.001). The cost burden of PC hospitalizations resulting in the death of patients and HD increased from 2005 to 2011. In 2011, the total hospitalization charges were 501.75 million dollars and 526 million dollars for PC deaths and HD, respectively. Other yearly trends can be reviewed in Table 6.

**Table 6:** Yearly trends for pancreatic cancer patients

Year	2005	2006	2007	2008	2009	2010	2011	Total
Total pancreatic cancer cases (frequency)	70,068	71,496	79,336	85,770	83,479	91,099	93,275	574,523
Died (frequency)	7,878	7,921	7,753	8,427	7,920	7,628	7,568	55,095
Percentage of total pancreatic cancer patients who died during the hospitalization	11.20%	11.10%	9.80%	9.80%	9.50%	8.40%	8.10%	9.60%
Hospice (frequency)	7,161	8,157	9,017	10,501	10,519	11,337	11,804	68,496
Percentage of total pancreatic cancer patients who were discharged to hospice	10.20%	11.40%	11.40%	12.20%	12.60%	12.40%	12.70%	11.90%
Other (frequency)	55,028	55,418	62,566	66,843	65,039	72,134	73,904	450,932
Percentage of total pancreatic cancer patients discharged to other destinations	78.50%	77.50%	78.90%	77.90%	77.90%	79.20%	79.20%	78.50%
Metastasis (frequency)	36,850	36,121	41,861	43,655	42,555	46,090	46,615	293,747
Percentage of total pancreatic cancer patients who had metastatic disease	52.60%	50.50%	52.80%	50.90%	51%	50.60%	50%	51.10%
Palliative care evaluation (frequency)	2,014	2,781	2,982	4,768	7,312	9,326	11,144	40,327
Percentage of total pancreatic cancer patients who had a palliative care evaluation during the hospitalization	2.90%	3.90%	3.80%	5.60%	8.80%	10.20%	11.90%	7%
Deep vein thrombosis (frequency)	3,641	3,960	4,383	4,705	4,699	4,846	5,002	31,236
Percentage of total pancreatic cancer patients who had a deep vein thrombosis	5.20%	5.50%	5.50%	5.50%	5.60%	5.30%	5.40%	5.40%
Pulmonary embolism (frequency)	2,414	2,431	3,361	3,471	3,410	3,873	3,979	22,939
Percentage of total pancreatic cancer patients who had a pulmonary embolism	3.40%	3.40%	4.20%	4%	4.10%	4.30%	4.30%	4%
Mechanical ventilation (frequency)	1,828	2,021	25,444	2,657	2,966	2,827	2,934	17,777
Percentage of total pancreatic patients who received mechanical ventilation	2.60%	2.80%	3.20%	3.10%	3.60%	3.10%	3.10%	3.10%
Mean Charlson Comorbidity Index (CCI)	5.04	5.08	5.25	5.45	5.54	5.51	5.59	5.37

## Discussion

The number of PC hospitalizations has been on the rise with each passing year. However, deaths in hospitals have decreased over the same period. HD gradually increased during the same period. The utilization of palliative care as a resource increased dramatically during the same period. However, during the same time, we did not notice a decrease in the percentage of patients who received aggressive care. Being a female was associated with fewer in-hospital deaths and increased HD.

These findings are consistent with the increasing incidence of PC in general [2]. Previous studies have noted the increased use of hospice over the years. However, it was also noted that an increase in hospice usage did not necessarily offset aggressive care near EOL [24]. Decreased odds of inpatient deaths and aggressive EOL care in females have been noted in a previous study [25].

The proportion of patients with metastatic PC dropped from 2005 to 2011. This is an indirect indicator that the hospitalizations of patients with incurable diseases have decreased over time. Potential explanations for this decrease are multifactorial. Patients with PC were either diagnosed in earlier stages, making them suitable surgical candidates, or an increased number of patients receiving palliative care and hospice utilization in the later years prevented re-admissions of patients with incurable metastatic disease. Given the challenges of diagnosing pancreatic cancer early, the latter explanation is more likely.

The incidence of DVT and PE increased slightly from 2005 to 2011. Oral anticoagulation during hospitalization was associated with decreased mortality and an increase in HD. Despite receiving IVCF, the HD percentage was much higher than the rest of the PC patients. This may suggest that patients who received IVCF insertion had advanced disease with other comorbidities, excluding them from receiving anticoagulation. Given the shortened anticipated survival in patients with advanced cancers, IVCF does not improve survival and may even negatively affect the quality of life [26].

The proportion of cases that received mechanical ventilation (MV) increased slightly from 2005 to 2011. This is an indirect metric to measure aggressive ICU level of care. More than half of these patients died during hospitalization. Not surprisingly, cases that received MV had a decreased HD rate compared to other PC patients (7.8% vs. 12.1%, p<0.001). This could indicate patient/family preference to pursue aggressive care. Previous studies have shown that patients disenrolling from hospice have higher healthcare usage and expenditures with increased ED visits, hospitalizations, and ICU admissions. The mortality rate is exceedingly high (57%) in such patients [27].

Although the data we reported represent an improvement in the utilization of palliative care, HD, and inpatient death numbers, the number of patients receiving aggressive interventions such as MV still remains high. Moreover, these interventions are associated with worse outcomes. Ideally, patients with terminal PC should have PCE earlier in the disease course and establish a reasonable and realistic plan of care. This would enable patients to have a better understanding of their prognosis and help them pursue an appropriate plan of care. This will also likely help decrease the number of hospitalizations and aggressive interventions near EOL. Finally, it should also be mentioned that despite the best efforts from healthcare providers, ultimately the direction of care and the level of aggressiveness in care is determined by patients’ personal wishes. This aspect of patient preference can be hard to quantify and account for when analyzing the quality-of-care metrics in cancer patients. 

Strengths of the study

The NIS database consists of cases from 48 states and includes patient populations from diverse socioeconomic backgrounds and demographics, making the results obtained more generalizable to the whole population of the United States. It has a large sample size, thereby making it possible to detect statistically significant differences among different variables. Since our study includes data from multiple years, it allowed us to observe trends and changes across time.

Limitations of the study

NIS is an administrative database, and thus clinical information such as histopathology, cancer stage, details of lab values, surgical reports, and imaging studies was not available. Longitudinal follow-up could not be done for the patients after discharge from the hospital. Information about outpatient treatment and care was not available. Patient and family preferences about the level of aggressiveness of care could not be ascertained from this database. We could not determine if the patients disenrolled from hospice before admission. The data used for this study is from more than a decade ago. This is because the method of reporting discharges changed in 2011, making it difficult to distinguish HD (home or facility) from routine discharges and discharge to a nursing home in the data from the later years. Despite utilizing older data, this analysis provides valuable insights into the trajectory of outcomes for PC patients through the years. 

## Conclusions

Over the years, hospitalizations for PC have increased, but inpatient deaths have decreased, with increased utilization of hospice as palliative care consultation has markedly increased. However, a corresponding decrease in indicators of aggressive care still seems to be lacking. With an increase in median survival times of PC patients, an increase in the incidence of PC-related complications is expected. Pursuing aggressive interventions for these complications is not always associated with better outcomes and, on the contrary, may be associated with worse outcomes in some cases. Thus, early evaluation of PC patients by palliative care is desirable, preferably in the outpatient setting. This would enable discussions about prognosis and goals of care in a less stressful environment, and help the patients come up with a plan of care that aligns with their values and is also medically appropriate. This would help keep PC patients out of the hospital and prevent aggressive interventions near EOL. However, patient preference is a big factor that ultimately determines the plan of care. Until better-targeted treatments and screening options become available for PC, mortality will remain high. The trends that we found in our analysis are encouraging, and surely signify a step in the right direction.

## Appendices

**Table 7:** Appendix 1. International Classification of Disease 9th Revision (ICD-9) codes relevant to the study

ICD-9 codes	Diagnosis/procedure
157.0 to 157.9	Pancreatic cancer
196.0 to 196.9	Secondary and unspecified malignant neoplasm to lymph nodes of head, face, neck, intrathoracic, intraabdominal, axillary, upper limb, inguinal, lower limb, intrapelvic, multiple sites, unspecified
197.0 to 197.8, 198.0 to 198.889, 199.0	Secondary malignant neoplasm of lung, mediastinum, pleura, other respiratory organs, small intestine/duodenum, colon/rectum, retroperitoneum/peritoneum, liver, other digestive organs/spleen, kidney, other urinary tract organs, skin, brain, spinal cord, other nervous system metastases, bone/marrow, ovary, adrenal glands, breast, genitals, other specified sites, disseminated without specific site
537.0 to 537.9	Gastroduodenal complications including hypertrophic pyloric stenosis, gastric diverticulum, duodenal ileus, other obstruction of the duodenum, fistula to stomach or duodenum, gastroptosis, hourglass stricture/stenosis of the stomach, pylorospasm, angiodysplasia of stomach/duodenum, dieulafoy lesion of stomach/duodenum, other specified and unspecified diseases of stomach and duodenum
575.0 to 575.9, 576.0 to 576.9	Gall bladder-related complications including acute, unspecified, chronic, acute and chronic cholecystitis; obstruction, hydrops, perforation, fistula of gall bladder; cholesterolosis of the gall bladder, other specified and unspecified complications of gall bladder; postcholecystectomy syndrome, cholangitis; obstruction, perforation, fistula of the bile duct; spasm of the sphincter of Oddi, other specified and unspecified complications of biliary tract
577.0 to 577.9	Pancreas-related complications including acute, chronic pancreatitis; cyst/pseudocyst of the pancreas, other specified and unspecified complications of pancreas
567.0 to 567.9, 568.89, 568.9	Peritonitis, other specified diseases of the peritoneum, unspecified disease of peritoneum
453.40 to 453.42, 453.2, 453.81, 453.83, 453.9	Venous thromboembolism (VTE) of the lower extremity, other VTE IVC, VTE of the upper extremity, other VTE of unspecified site
415.11, 415.13, 415.19	Pulmonary embolism: iatrogenic and infarction, saddle PE of the pulmonary artery, other PE and infarction
296.20 to 296.36, 311	Depression
799.4	Cachexia
276.50 to 275.52, 785.50 to 785.59	Hypovolemia, shock
584.5 to 584.9	Acute kidney injury
521.1 to 521.9	Biopsy of pancreas
542.1 to 542.9	Laparoscopy, biopsy of abdominal wall/umbilicus, peritoneum, closed/needle biopsy of the intraabdominal mass, peritoneal lavage, other diagnostic procedure of abdomen
992.5	Chemotherapy injection
288.00 to 288.09	Neutropenia
525.1 to 525.9, 526, 527	Pancreatic surgeries including proximal, distal, radical subtotal, other, total pancreatectomy; Whipple procedure/radical pancreaticoduodenectomy
518.7	Endoscopic insertion of biliary stent
518.1 to 518.9	Sphincter of Oddi-related procedures including sphincterotomy, sphincteroplasty, dilation of the ampulla of Vater, sphincterotomy/papillotomy, endoscopic nasobiliary drain tube, removal of stone, other operation on sphincter of Oddi
99.00 to 99.09	Transfusion of blood products including autologous blood, other whole blood, packed cells, components, platelets, coagulation factors, other serum, blood expander, other substances
9910	Injection/infusion of thrombolytic agent
991.9	Injection of anticoagulant
38.7	Interruption of Inferior vena cava/IVC filter
00.17,	Infusion of vasopressor
967.0 to 967.2	Ventilator support
V58.61	Long-term use of anticoagulants
39.95	Hemodialysis
99.15	Parenteral infusion of nutritional substances
V66.7	Encounter for palliative care
